# A method to obtain the thermal parameters and the photothermal transduction efficiency in an optical hyperthermia device based on laser irradiation of gold nanoparticles

**DOI:** 10.1186/1556-276X-9-441

**Published:** 2014-08-27

**Authors:** Cristina Sánchez López de Pablo, José Javier Serrano Olmedo, Alejandra Mina Rosales, Norma Ramírez Hernández, Francisco del Pozo Guerrero

**Affiliations:** 1 Centre for Biomedical Technology (CTB), Universidad Politécnica de Madrid (UPM), Campus de Montegancedo, Pozuelo de Alarcón, 28223 Madrid, Spain; 2 Biomedical Research Networking Center (CIBER), C/ Monforte de Lemos 3-5, Pabellón 11, 28029 Madrid, Spain

**Keywords:** Gold nanoparticles, Laser irradiation, Optical hyperthermia, Photothermal transduction efficiency, Temperature curves, Thermal parameters

## Abstract

Optical hyperthermia systems based on the laser irradiation of gold nanorods seem to be a promising tool in the development of therapies against cancer. After a proof of concept in which the authors demonstrated the efficiency of this kind of systems, a modeling process based on an equivalent thermal-electric circuit has been carried out to determine the thermal parameters of the system and an energy balance obtained from the time-dependent heating and cooling temperature curves of the irradiated samples in order to obtain the photothermal transduction efficiency. By knowing this parameter, it is possible to increase the effectiveness of the treatments, thanks to the possibility of predicting the response of the device depending on the working configuration. As an example, the thermal behavior of two different kinds of nanoparticles is compared. The results show that, under identical conditions, the use of PEGylated gold nanorods allows for a more efficient heating compared with bare nanorods, and therefore, it results in a more effective therapy.

## Background

Nanoscale materials have been broadly studied in recent years, thanks to their unique optical properties and their great potential in the development of biomedical applications. One of the most interesting areas is the use of plasmonic nanoparticles to enhance the diagnostic and treatment methods available for cancer. In this field, authors such as Letfullin and co-workers have recently described the optical properties, the kinetics of heating and cooling, and the spatial distribution of temperature of this kind of nanoparticles, providing a better understanding of these processes [1-3].

Specifically, rod-shaped gold nanoparticles, known as gold nanorods (GNRs), have a great potential because of their large light absorption and scattering cross sections in the near-infrared (NIR) region [4]. Thanks to this optical behavior, GNRs are able to transform the absorbed energy into localized heat. This optical effect is used to develop cancer therapies as photothermal tumor destruction either by direct enough increase of temperature or indirectly by co-adjuvant drugs, at the same time delivered by the particle, or already present and activated by the heating [5-8].

Our research group has recently developed an optical hyperthermia device based on irradiation of GNRs with a continuous wave (CW) laser in order to induce *in vitro* death of human brain astrocytoma cells (1321 N1) [9]. Unlike many high-energy pulsed lasers that generally lead to particle structure changes and ablation in a very short time, CW lasers allow heat dissipation from particles to surrounding medium (via phonon-phonon relaxation), so they are an appropriate choice in order to use the produced heat for the cure of cancer [10].

The effectiveness of the developed method was determined by measuring changes in cell viability after laser irradiation of cells in the presence of GNRs. In accordance to other results in comparable experiments [11-13], ours indicated that continuous laser irradiation in the presence of the particles induced a significant decrease in cell viability, while no decrease in cell viability was observed with laser irradiation or incubation with GNRs alone. Due to the limited capacity of laser penetration in tissues, this method could be used in clinical practice as an additional aid to surgery for removing brain tumors completely.

After this proof of concept, our objective was focused in getting a better understanding about the working principles and physical behavior of optical hyperthermia devices. It is not very common to find studies including a comprehensive characterization about the global phenomena in optical hyperthermia systems. Moreover, although now there are a huge variety of noble metal nanoparticles that can be used to carry out this kind of therapy, an absolute control about their behavior still does not exist. Therefore, it is necessary to develop a series of characterization and modeling processes to increase the effectiveness of the hyperthermia treatments, thanks to the prediction of the system response.

With this aim, a method to calculate the thermal parameters of the system and the photothermal transduction efficiency for different kinds of nanoparticles has been developed. This method, which allows an easy and effective thermal characterization and so predicts the thermal behavior of the system, is not only valid for our device but also for any kind of optical hyperthermia system.

## Methods

### Optical hyperthermia device

The CW laser (MDL H808, PSU-H-LED power source, Changchun New Industries, Changchun, China) works at 808 nm, with a maximum output power of 5 W, beam height from base of 29 nm, beam diameter at aperture of 5 to 8 mm, and dimensions of laser head of 155 × 77 × 60 mm^3^.

We have used two different kinds of commercial GNRs in order to compare their photothermal transduction efficiency. Both are tuned to the laser source and have their maximum surface plasmon resonance (SPR) at 808 nm (longitudinal band).

The first commercial GNRs used are bare GNRs (B-GNRs) A12-10-808-100 Nanorodz (Nanopartz, Salt Lake City, UT, USA). B-GNRs are dispersed in deionized water (DI-H_2_O) with <0.1% ascorbic acid and <0.1% cetyltrimethylammonium bromide (CTAB) surfactant capping agent. B-GNRs have an axial diameter of 10 nm and a length of 41 nm.

**Figure 1:**
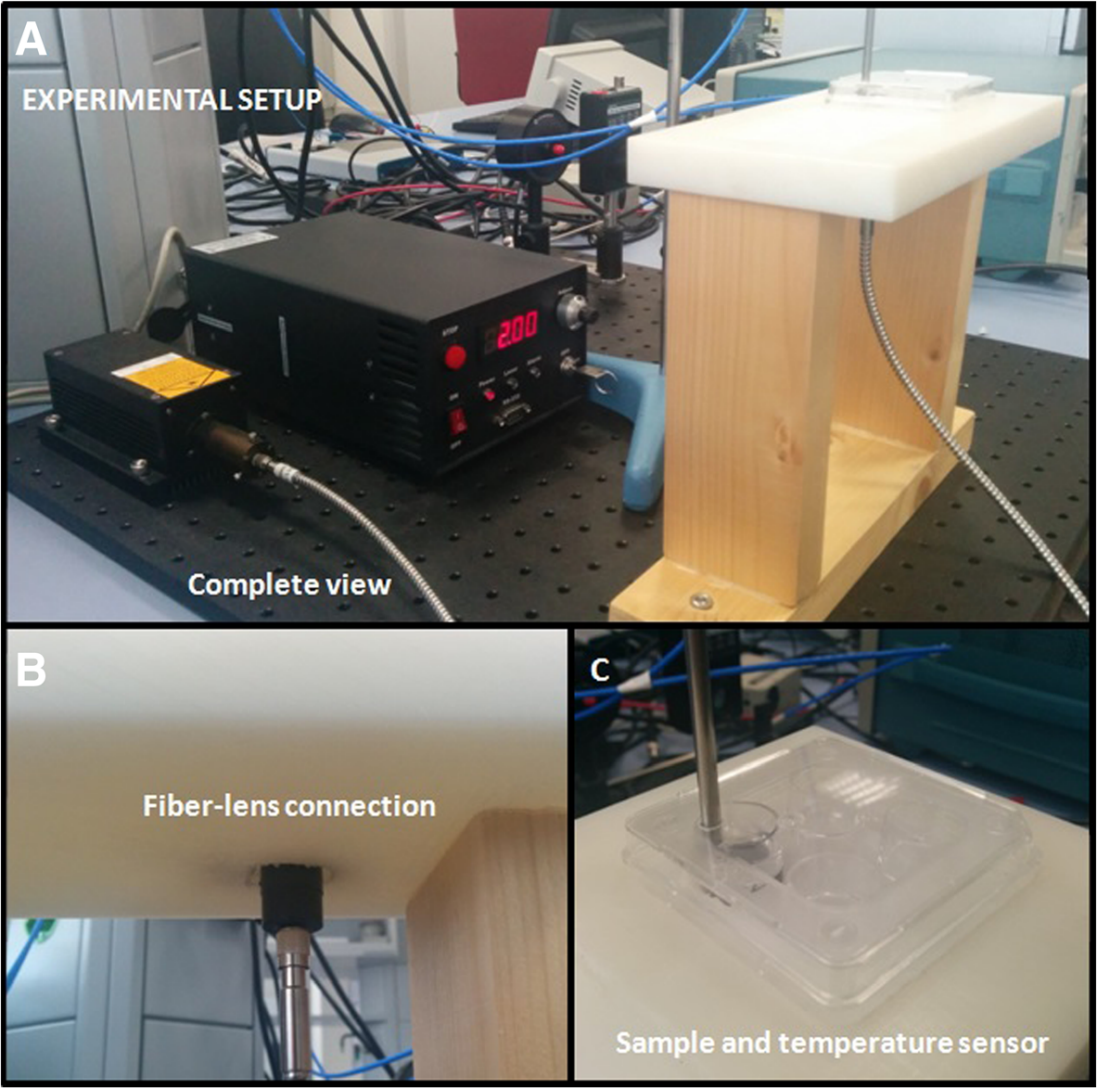
Experimental setup: complete view (A), fiber-lens connection details (B), and sample and temperature sensor details (C).

The other commercial GNRs used are PEGylated GNRs (PEG-GNRs) PEG-10-808-50 (Nanoseedz, China). PEG-GNRs are functionalized by thiol-terminated methoxypoly(ethylene glycol) (mPEG-PH) and are also dispersed in DI-H_2_O. The dimensions of PEG-GNRs are equal to the dimensions of B-GNRs (axial diameter = 10 nm, length = 41 nm).The laser is connected to the system via a multimode optical fiber with a core diameter of 600 μm, a length of 1.5 m, and a power transmission of 90% to 99% (600-μm MM fiber, Changchun New Industries, China). The laser light from the fiber irradiates the samples through a collimation lens (78382, Newport Corporation, Irvine, CA, USA), which is in direct contact with a 4-well plate containing the samples, which have a total volume of 500 μl, and is located on a Teflon support. A temperature sensor (F100 Precision Thermometer, Automatic Systems Laboratories, Redhill, UK) is fixed vertically with the aid of a tripod stand and a burette clamp and remains in contact with the samples during the experiments (Figure 1).

### Thermal parameters

**Figure 2:**
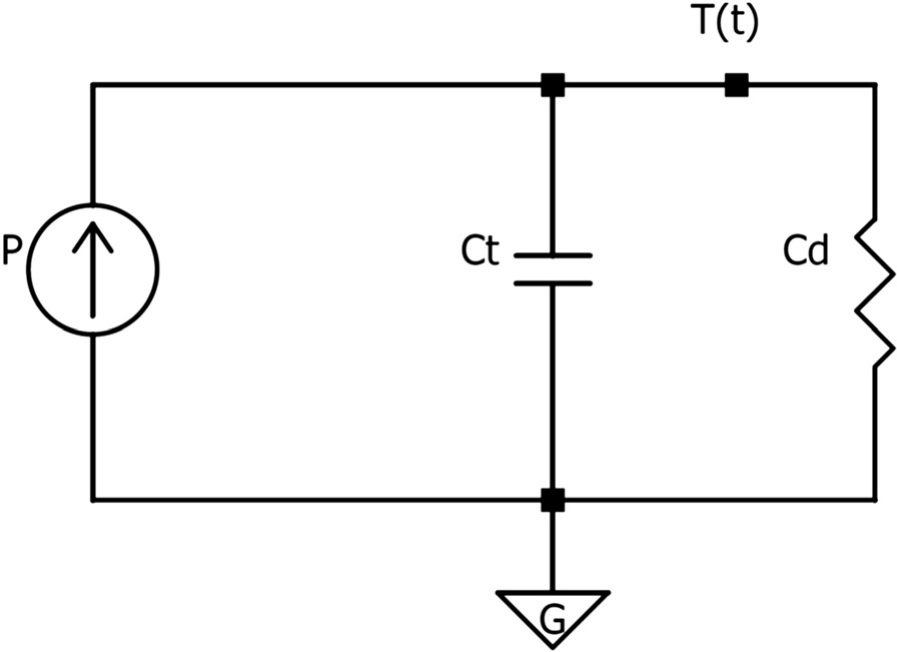
Electrical equivalent circuit used to obtain the thermal parameters of the optical hyperthermia device.

In order to determine the parameters that characterize and describe the thermal behavior of our hyperthermia device, it is needed to develop a thermal model, which can be raised from the resolution of an equivalent electric circuit (Figure 2).

In this circuit, *P* is the delivered power, *T*(*t*) is the sample temperature which is time dependent, and *C*_*d*_ (W/K) and *C*_*t*_ (J/K) are the thermal conductance and the thermal capacitance of our experimental enclosure, respectively.

Solving the circuit, we can formulate the equation that describes the power distribution, obtaining that the delivered power (*P*) is equal to the sum of the stored power in the capacitor (*P*_s_) and the dissipated power in the resistor (*P*_d_):

(1) $$\begin{array}{ll} P & =P_{s}+P_{d}\\ & =C_{t}\frac{d\left(T\left(t\right)-T_{\text{ref}-m}\right)}{\mathrm{dt}}+\left(T\left(t\right)-T_{\text{ref}-m}\right)C_{d} \end{array}$$

In this equation, *T*_ref *- m*_ is the reference temperature (the subscript *m* refers to the thermal model), that is to say, the initial temperature of our sample before the laser irradiation that should match the environment temperature. Once the capacitor is charged, the temperature had reached their maximum value (*T*_max *- m*_), also called stabilization temperature, which remains constant until the end of the irradiation. Under this condition, we can rewrite the previous equation removing the time dependence as

(2) $$P=\left(T_{\text{max}-m}-T_{\text{ref}-m}\right)C_{d}=\Delta T_{m}C_{d}$$

In a conventional experiment of optical hyperthermia, a laser source irradiates a sample containing a colloidal suspension of GNRs which act as little heat sources. In the proposed model, the GNRs are replaced by an electric resistor (*R*) which is connected to a voltage source (*V*) so that we dispose of a single heat source delivering a known power:

(3) $$P=\frac{V^{2}}{R}$$

The resistor heats up the sample until the stabilization temperature (*T*_max - *m*_) is reached, and then, the voltage sample is shut off and the resistor is immediately removed from the sample in order to obtain the cooling curves (which correspond to the discharge curves of the capacitor) that characterize our experimental enclosure without the influence of the resistor that is still kept warm.

By adjusting these cooling curves to the corresponding decreasing exponential equation, we can obtain the cooling time constant, which depends on the thermal capacitance and the thermal conductance of our system:

(4) $$t\;\left(s\right)=\frac{C_{t}\;\left(J/K\right)}{C_{d}\;\left(W/K\right)}$$

Thus, from a known power and from the values of *τ* and Δ*T*_*m*_ experimentally obtained, we can calculate the thermal parameters (i.e., thermal capacitance and thermal conductance) that characterize the sample enclosure of the described optical hyperthermia device.

In this case, we chose a resistor of 15.2 Ω, the voltage source values were 1.5, 2.0, and 2.5 V, and the tested sample volumes were 500, 750, and 1,000 μl. We obtained three heating and cooling temperature curves for each possible configuration.

### Photothermal transduction efficiency

From the Mie theory and taking into account different parameters such as the nanoparticle size and shape, the refractive index of the surrounding medium, and the laser wavelength, authors such as Zharov describe the optimal conditions for nanoparticles to obtain effective laser heating in optical hyperthermia applications [14].

On the other hand, we can find in the literature advanced models that completely describe the heat transfer behavior from the surface of nanoparticles presenting the heat sources produced by nanoparticles in the spherical volume of biological tissue [15,16]. These methods allow for predicting the complete thermal response for applications to future cancer therapies as nanophotothermolysis and nanophotohyperthermia, but we propose a simpler approach in order to rapidly compare the photothermal response of nanoparticles in optical hyperthermia devices to be able to select those nanoparticles that allow us to obtain better results in each planned therapy.

With the aim of obtaining the photothermal transduction efficiency of the two different kinds of GNRs used as heat sources in the described optical hyperthermia device, it is needed to measure the time-dependent heating and cooling temperature curves of the irradiated samples containing the suitable colloidal dispersion of each kind of GNRs. Roper et al. [17] determine the energy balance used to describe this process (Equation 5):

(5) $$\sum_{i}m_{i}C_{\mathrm{pi}}\frac{\mathrm{dT}}{\mathrm{dt}}={\dot{Q}}_{I}+{\dot{Q}}_{0}-\;{\dot{Q}}_{\text{ext}}$$

In the previous expression (Equation 5), *m* and *C*_*p*_ are the mass and the heat capacitance of each component of the irradiated sample, respectively, *T* is the temperature of the sample, *Q*_*I*_ is the calorific energy that GNRs generate (energy source), *Q*_0_ is the baseline energy of the sample (represents the temperature rise of the sample due to the direct heating of the laser source), and *Q*_ext_ represents the energy flux transmitted out of the irradiated area.

The term *Q*_*I*_ represents the heat that is generated due to the electron-phonon relaxation of plasmons in the surface of GNRs that takes place because of the irradiation of the particles at the SPR wavelength *λ*:

(6) $${\dot{Q}}_{I}=I\left(1-10^{-A_{\lambda }}\right)\eta$$

In this expression (Equation 6), *I* is the power of the incident laser irradiation after the attenuation due to the different optical elements in the light path, *η* is the photothermal transduction efficiency (the parameter we want to calculate) that denotes a value for the efficacy of GNRs converting the incident light that interacts with them into thermal energy, and *A*_*λ*_ is the optical density (also called absorbance) of the sample (colloidal dispersion) at the irradiation wavelength.

The outgoing heat flux can be considered linearly proportional to the thermal driving force, with a heat transfer coefficient, *h*, as proportionality constant:

(7) $${\dot{Q}}_{\mathrm{ext}}=\mathrm{hA}\left(T-T_{\text{ref}}\right)$$

Therefore, the outgoing heat rate could be described using a lineal model with respect to the temperature, which results in the following equality when there is no incident laser light over the sample:

(8) $$\sum_{i}m_{i}\;C_{p,i}\frac{\mathrm{dT}}{\mathrm{dt}}=-\mathrm{hA}\left(T-T_{\text{ref}}\right)$$

In the previous equations (Equations 7 and 8), *T*_ref_ is the environment temperature and *A* is the irradiated area that the heat flux crosses toward the non-irradiated area. On the one hand, following this model, we can state that the part of the thermal cycle that defines the cooling of the sample exponentially depends on the time, and thereby, it is possible to determine the characteristic thermal time constant of the system by finding the exponential that adjusts the temperature curve. On the other hand, the heat transfer coefficient is inversely proportional to this time constant and could be defined as it is shown in the next expression:

(9) $$h=\frac{\Sigma_{i}m_{i}C_{p,i}}{\tau A}=\frac{C_{t}}{\tau A}$$

Once we know the heat transfer coefficient, it can be used to calculate the amount of energy that the sample accumulates or losses, from the temperature evolution.

When the irradiated sample reaches the equilibrium temperature (*T*_max_), the incoming heat flux should be equal to the outgoing heat flux (toward the non-irradiated area of the sample), and therefore, *T*_max_ is directly proportional to the optical power absorbed by GNRs:

(10) $$\begin{array}{l} {\dot{Q}}_{I}+{\dot{Q}}_{0}-\;{\dot{Q}}_{\text{ext}}=0\;\to \;{\dot{Q}}_{I}+{\dot{Q}}_{0}={\dot{Q}}_{\mathrm{ext}}\\ {\dot{Q}}_{I}+{\dot{Q}}_{0}=\mathrm{hA}\left(T_{\text{max}}-T_{\text{ref}}\right) \end{array}$$

Therefore, we can solve the value of the photothermal transduction efficiency from the previous equations as follows:

(11) $$\eta =\frac{\mathrm{hA}\left(T_{\text{max}}-T_{\text{ref}}\right)-{\dot{Q}}_{0}}{I\left(1-10^{-A_{\lambda }}\right)}$$

Finally, if we rewrite the equation as a function of *C*_*t*_ and rename *T*_max_ - *T*_ref_ = Δ*T*, we obtain the following expression, in which all the terms can be gotten from the calculation of the thermal parameters and the corresponding temperature curves for a fixed laser power irradiation:

(12) $$\eta =\frac{C_{d}\Delta T-{\dot{Q}}_{0}}{I\left(1-10^{-A_{\lambda }}\right)}$$

In this case, we have estimated the value of *η* for the two kinds of nanorods described before (B-GNRs and PEG-GNRs). In both cases, the calculation was carried out from the temperature curves of three equal samples of water-dispersed GNRs with *A*_*λ*_ = 1 (which corresponds with a concentration of 36 μg/ml) in order to obtain Δ*T* and from the temperature curves of three equal samples of deionized water to obtain *Q*_0_. All samples were irradiated with a laser power average of 2.0 W, and their volume was 500 μl.

## Results and discussion

### Thermal parameters

**Figure 3:**
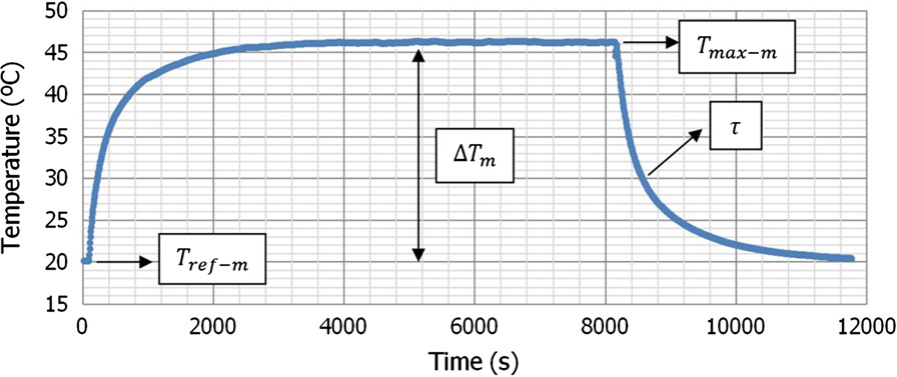
Temperature curve of heating, stabilization, and cooling, obtained from the thermal model and the proposed procedure.

As described previously, we obtained three temperature curves of heating, stabilization, and cooling for each proposed case. Figure 3 shows schematically the shape of these curves and the parameters that we can get from them.

**Figure 4:**
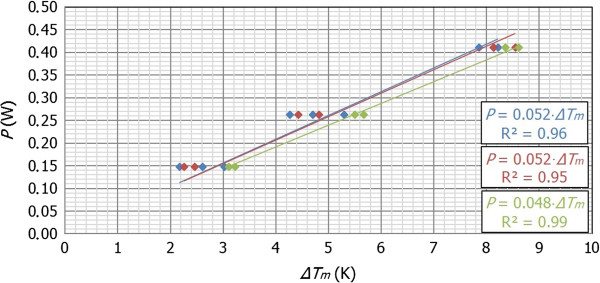
**Relation between *P *(W) and** Δ***T***_***m ***_**(K).** Lineal fits for each tested value: 500 μl (blue), 750 μl (red), and 1,000 μl (green), whose values of thermal conductance are 0.052, 0.052, and 0.048 W/K, respectively. *R*^2^ is the average squared error of each fit.

We know that the thermal conductance is obtained from the data of power and temperature variation so that *C*_*d*_ *= P /* Δ*T*_*m*_. Therefore, if we represent the value of *P* graphically as a function of Δ*T*_*m*_, it is possible to make a lineal fit in order to obtain the desired value of thermal conductance as shown in Figure 4.As it can be observed in Figure 4, the values of thermal conductance are pretty similar for the three considered volumes. This behavior is consistent with the fact that the thermal conductance is an intensive magnitude, and therefore, it does not depend on the volume of the sample but on the global thermal properties of the considered system.

Then, the thermal conductance of our system could be estimated from the average of the thermal conductances obtained for each volume: *C*_*d*1_ (500 μl) = 0.052 W/K, *C*_*d*2_ (750 μl) = 0.052 W/K, and *C*_*d*3_ (1,000 μl) = 0.048 W/K so that *C*_*d*_ *≈* 0.051 W/K.

**Figure 5:**
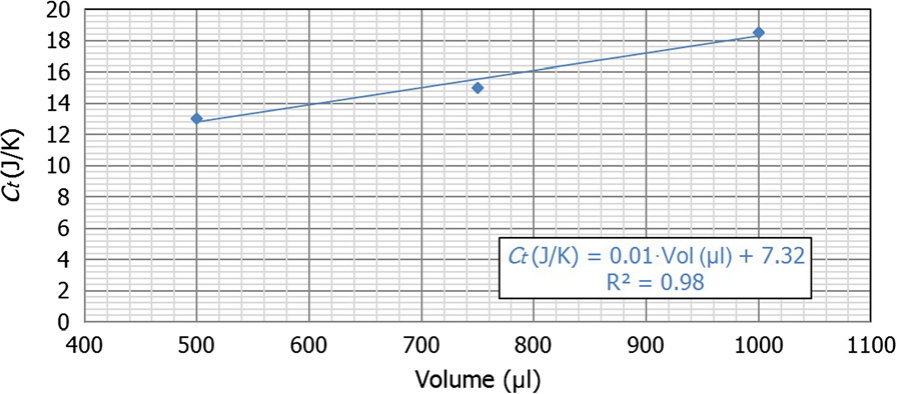
**Thermal capacitance values *C***_***ti ***_**(J/K) as a function of the sample volume (Vol).***R*^2^ is the average squared error of the fitted line.

Then, Table 1 shows the average values of *τ*_*i*_ obtained for each tested volume and the associated values of thermal capacitance *C*_*ti*_ (J/K), and Figure 5 represents graphically this evolution of the thermal capacitance as a function of the volume.

**Table 1:** **Values of the average time constant***τ*_***i ***_**and thermal capacitance for each tested volume**

**Volume (μl)**	*τ*_***i***_	***C***_***ti ***_**(J/K)**
500 (*i* = 1)	256.05	13.06
750 (*i* = 2)	295.15	15.05
1,000 (*i* = 3)	363.72	18.55

In this case, we can observe that the values of thermal capacitance (expressed in J/K) have an increasing lineal evolution depending on the volume. This effect is consistent with the fact that the thermal capacitance is an extensive property, because their value depends on the amount of substance. Indeed, this parameter is defined as the ratio between the amount of thermal energy delivered to a body and the temperature change that the body experiments so that the greater is the volume, the lower is the experimented temperature change for a fixed amount of energy, and therefore, the greater is the value of the thermal capacitance.

### Photothermal transduction efficiency

Once the thermal parameters have been calculated, to estimate the value of *η*, it is needed to know the percentage of the irradiation power (*P*_LASER_) incident on the sample (*I*) by taking into account the losses due to the light path through the optical elements between the output of the fiber and the sample. In this case, in the light path, there are a collimated lens and a 4-well plate. The lens has two faces and a percentage of losses of about 3%, and the 4-well plate adds a percentage of losses near 10% according to the manufacturers. Therefore, the total amount of losses is about 16%, and then, the incident power could be expressed as *I* = (1 - 0.16) *P*_LASER_.

**Figure 6:**
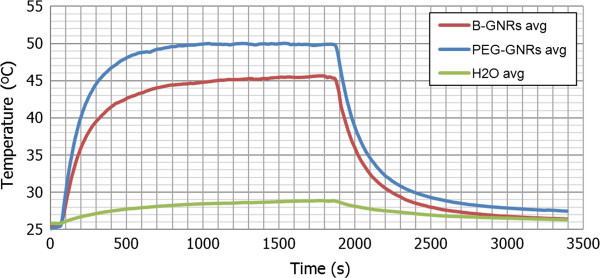
**Temperature curves of heating, stabilization, and cooling (average values).** Obtained for the irradiated samples of B-GNRs, PEG-GNRs, and deionized water (500 μl, *A*_*λ*_ *=* 1, *P*_LASER_ *=* 2.0 W).

Figure 6 shows the temperature curves obtained for the irradiated samples of B-GNRs, PEG-GNRs, and deionized water, *P*_LASER_ = 2.0 W, and Table 2 shows the parameters (average values) obtained from these temperature curves and the photothermal transduction efficiency that has been calculated from them according to Equation 12 (average values from three equal measures in each case).

**Table 2:** **Parameters and photothermal transduction efficiency obtained from samples of B-BNRs and PEG-GNRs (500 μl, *A***_***λ***_ ***=*** **1, *P***_**LASER**_ ***=*** **2.0 W)**

***I *** **(W)**	**GNRs**	Δ ***T *** **(°C)**	***Q***_**0 **_**(W)**	*η***(%)**
1.68	B-GNRs	20.5	0.20	56.0
	PEG-GNRs	25.1		71.4

**Figure 7:**
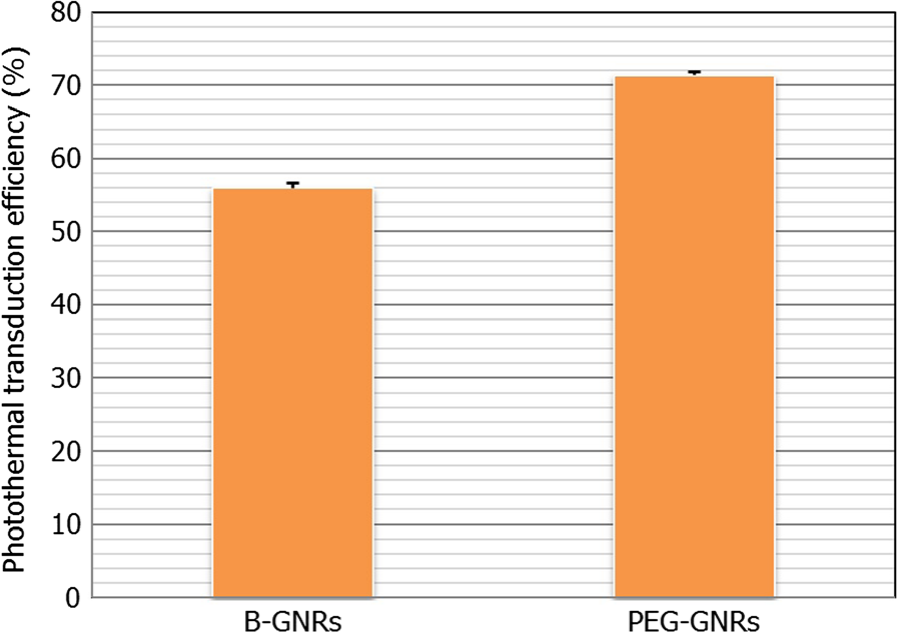
**Estimated values of *η *for each one of the tested kinds of GNRs: B-GNRs and PEG-GNRs.** Presented as the average value (from the three temperature curves obtained for each case) ± the standard deviation.

Finally, Figure 7 shows the estimated values of *η* for each one of the tested kinds of GNRs (B-GNRs and PEG-GNRs) presented as the average value (from the three temperature curves obtained for each case) ± the standard deviation.

From the observation of the previous graph, we can affirm that under the established conditions of experimentation, the photothermal transduction efficiency of PEG-GNRs is about 15% to 16% higher than in B-GNRs. Thus, under the described working conditions, the use of PEG-GNRs allow for a more efficient heating of the sample, and therefore, the use of this kind of nanoparticles results in a more effective therapy.

## Conclusions

The estimation of the thermal parameters and the photothermal transduction efficiency allows us to compare the thermal behavior of different kinds of nanoparticles under similar working conditions. Moreover, it could be also useful to compare the thermal response of different kinds of nanoparticles under different working conditions as, for example, concentration, optical density, dispersion media, or sample holder. Therefore, the photothermal transduction efficiency is needed to determine the optimal conditions depending on each considered case.

To summarize, we can say that, from a series of input data to the system, as the power of irradiation and the optical density of the used nanoparticles, it is possible to calculate the photothermal transduction efficiency of these particles using the thermal parameters of the system and the temperature variation of the samples. Therefore, it is possible to determine, for any kind of gold nanoparticles (or other noble metals) with their peak of absorption syntonized with the wavelength of irradiation, the percentage of the optical power that interacts (absorption + scattering) with the sample that really becomes in a temperature increasing. The higher the value of this parameter, the higher the efficiency of the designed optical hyperthermia treatment, and so, if we know the value of this parameter previously, we could select those nanoparticles that allow us to obtain better results in the designed therapy.

## Competing interests

The authors declare that they have no competing interests.

## Authors’ contributions

All authors contributed equally to this work. All authors read and approved the final manuscript.
